# Yeast cell wall derivatives as a potential strategy for modulating oral microbiota and dental plaque biofilm

**DOI:** 10.3389/froh.2025.1543667

**Published:** 2025-02-13

**Authors:** Torsten P. M. Scheithauer, Isabela M. Fernandes de Oliveira, Michel Ossendrijver, Elodie Dehay, Michelle van der Wurff, Hakim Rahaoui, Nathalie Ballet, Bart J. F. Keijser

**Affiliations:** ^1^ Department of Microbiology & Systems Biology, TNO, Leiden, Netherlands; ^2^ Scientific Affairs, Gnosis by Lesaffre, Marcq-en-Baroeul, France; ^3^ Discovery & Front-End Innovation, Lesaffre Institute of Science & Technology, Lesaffre International, Marcq-en-Baroeul, France; ^4^ Department of Preventive Dentistry, Academic Centre for Dentistry Amsterdam, University of Amsterdam and Vrije Universiteit Amsterdam, Amsterdam, Netherlands

**Keywords:** oral microbiota, periodontitis, yeast, postbiotic, biofilm, oral health

## Abstract

**Introduction:**

Derivatives from *Saccharomyces cerevisiae* yeast including yeast extracts and yeast cell walls are sustainable sources of valuable nutrients, including dietary fibers and proteins. Previous studies have shown that certain components from these yeast derivatives can inhibit the growth of harmful intestinal bacteria and promote the growth of beneficial bacteria. However, the effects of yeast derivatives on oral health have not yet been investigated.

**Methods:**

An *in vitro* oral biofilm model was employed to examine the impacts of yeast derivatives on the oral microbiota and their potential benefits for maintaining oral homeostasis. The model incorporated dental plaque donor material from both healthy and periodontitis diagnosed individuals. Biofilm formation, density, and microbial composition were quantified. Additionally, the production of short-chain fatty acids in the biofilm supernatants was measured.

**Results:**

Yeast extracts had only minor effects on oral biofilm formation. In contrast, yeast cell wall derivatives, which are rich in polysaccharides such as beta-glucans and mannans, significantly reduced the density of the oral biofilms *in vitro*. This reduction in biofilm density was associated with an overall shift in the bacterial community composition, including an increase in beneficial bacteria and a decrease in the abundance of *Tannerella forsythia*, an important species involved in bacterial coaggregation and the development and maturation of the oral biofilm. Furthermore, the yeast cell wall derivatives decreased the production of short-chain fatty acids, including acetic and butyric acid. These findings were consistent across both healthy and periodontitis microbiomes.

**Conclusion:**

This study has demonstrated the potential of yeast cell wall derivatives to positively impact oral health by significantly reducing biofilm density, modulating the oral microbial composition, and decreasing the production of short-chain fatty acids. The observed effects highlight the promising applications of these yeast-based compounds as an approach to managing oral diseases. Further research is needed to fully elucidate the mechanisms of action and explore the clinical potential of yeast cell wall derivatives in promoting and maintaining oral health.

## Introduction

The oral cavity is a complex microbial ecosystem characterized by multiple niches with different biochemical, chemical, and physical properties, which are reflected by the unique microbial populations they harbor (1). Oral diseases are linked to shifts in the activity and composition of the oral microbiota. Tooth decay, also known as dental caries or cavities, is associated with microbial acid formation from the breakdown of sugars, dental demineralization, and gingival inflammation (gingivitis and periodontitis). These processes collectively contribute to host tissue and enamel damage and bone loss (2).

Several factors, including oral hygiene habits, host physiology and microbial ecology, contribute to maintaining a homeostatic oral ecosystem. However, poor oral health may lead to the accumulation and maturation of dental biofilms which are organized multispecies structures that based on the community ecology and potential pathogenicity might drive the development of oral diseases (3). Bacterial genera involved in the early colonization of the oral cavity, such as *Veillonella*, *Streptococcus, Actinomyces, Haemophilus* and *Neisseria* have been associated with a balanced microbiota and oral health (4, 5). Later stages of biofilm maturation involve the colonization by periodontal pathogens such as *Porphyromonas gingivalis* and *Tannerella forsythia* (6).

Periodontitis is a prevalent and serious oral health condition characterized by inflammation and infection of the supportive structures surrounding the teeth, including the gingiva (gums) and alveolar bone. If left untreated, it can result in tooth loss, systemic diseases and reduced quality of life. Gingivitis, the most common inflammatory condition of the gingival tissue often precedes the development of periodontitis (7). This condition is marked by a shift in the functionality and composition of dental biofilms, transitioning from saccharolytic to more proteolytic activity, often with an anaerobic nature (8). Periodontitis is associated with the accumulation and maturation of dental plaque characterized by an increased abundance of periodontal pathogens within the biofilms (9, 10).

Pro-, pre- and postbiotics are attractive alternatives for modulating the oral microbiota towards a homeostatic ecosystem (11). Prebiotics are defined as “a substrate that is selectively utilized by host microorganisms conferring a health benefit”, while probiotics are the “non-pathogenic microorganisms that exert health benefits to the host when administered in adequate quantity” (12, 13). Postbiotics, on the other hand, are defined as “a preparation of inanimate microorganisms and/or their components that confers a health benefit on the host” (14). These compounds include a wide range of substances such as enzymes, peptides, polysaccharides, cell wall components, and organic acids (15). Yeast extracts would be able to modulate the gut microbiota community composition (16). Derivatives from brewer's spent yeast are rich sources of beta-glucans and mannans, which are natural polysaccharides present in their cell wall and exhibit prebiotics/postbiotics properties (17). As previously reported, beta-glucans are able to inhibit the growth of intestinal pathogens and increase the abundance of beneficial bacteria (18). The polysaccharide mannan, composed primarily of mannose, is present in the outer lawyer of the cell wall and has various applications in biotechnology and the food industry, including as a food additive to improve intestinal disorders, directly influence microbial ecology, and reduce pathogen adherence (19). Several studies reported the benefic impact of these cell wall components on periodontitis and oral health in animal models, for instance, contributing to tissue repair, protective effects against bone loss, and improvements in the inflammatory profile (20–22). Yeast-based products, including probiotics and postbiotics, offer promising benefits for enhancing oral health through antimicrobial, immunomodulatory, and biofilm-inhibitory effects. Understanding and leveraging these interactions and derivatives can lead to improved strategies for preventing and managing oral diseases.

Human interventions for oral health can be expensive and laborious. *In vitro* models that replicate the diverse oral niches serve as powerful tools for screening new compounds and evaluating their potential benefits (23). The model employed in this study relies on the anaerobic cultivation of human oral microbial communities (microcosm) and the maturation of biofilms onto a solid surface. The growth conditions can be adjusted to develop either a more cariogenic or periodontal biofilm. By supplementing the cultivation medium with fetal calf serum, a more pronounced proteolytic biofilm was obtained. It is believed that serum provides a surrogate for gingival crevicular fluids, that are more abundant in inflamed dental pockets (9).

In this study, a gingivitis-prone *in vitro* model was used to examine the effects of yeast extracts and yeast cell wall formulations on the oral microbiota. Donor material from both healthy and periodontal disease diagnosed individuals was used in the study. The appearance, density, and microbial composition of the biofilms were investigated. Furthermore, the production of short-chain fatty acids (SCFAs) in the biofilm supernatants was also evaluated. This study demonstrates the beneficial effects of yeast cell wall extracts and their potential effect in improving oral health and preventing periodontitis.

## Material and methods

### *In vitro* oral biofilm model

For dental plaque biofilm sampling, volunteers were asked to abstain from brushing their teeth for a period of at least 18 h. The plaque was then collected from healthy participants through dental brushing and oral rinsing with a saline solution. The collected material was then used for culturing. Each of the three replicates involved a different group of volunteers, collected on different dates (*n* = 10 volunteers for each replicate). For each cultivation experiment of the microbiota of periodontitis patients, fresh dental plaque was collected from volunteers by students at the Utrecht University of Applied Sciences Dental Hygiene. The volunteers were clinically diagnosed with periodontitis, but their treatment only started after the time of sample collection. For each of the three replicates, a different group of volunteers was pooled, consisting of four, seven, and six volunteers, respectively. Due to the availability of diagnosed volunteers at the time of the experiment, the number of volunteers with periodontitis varied. All volunteers signed an informed consent, and the ethical approval for the study was granted by an internal ethical review board.

Immediately after collection, the dental plaque samples were transferred to tubes with peptone water supplemented with cysteine and stored in anaerobic jars for transport at room temperature. The next day, plaque samples were transferred to an anaerobic cabinet and pooled. The total amount of dental plaque biofilm collected from each donor were pooled, 40× diluted, and used for the culturing biofilm. The concentration of the starting material was not measured. The artificial saliva cultivation medium (modified McBain medium) was prepared fresh for each cultivation and pre-reduced under anaerobic conditions (24). The medium was supplemented with 2.5% sheep blood and 10% fetal calf serum, supporting outgrowth periodontal pathogens (9). Cultivation was performed in 12-well plates placed on a rotary platform (150 rpm) at 37°C under anaerobic conditions (Supplementary Material Figure S1). Glass coverslips, placed upright, were used as surfaces for biofilm attachment. The culture medium was refreshed every three days. The cultivation was conducted in three independent experiments, accommodating triplicates to assess the different parameters.

### Yeast-based products (YBP)

Nine yeast derivatives were tested, in which seven represent different yeast extract formulations (YBP-1 to −7) and two are yeast cell wall derivatives (YBP-8 and −9). These yeast derivatives were compared to whole live (LY-B) and inactivated yeast cells (IY). Information about the yeast strains, composition of the yeast products and suppliers are described in Supplementary Table 1. The yeast extracts YBP-1–7 were dissolved in demi water and sterilized by filtration. All other products were insoluble and added as a suspension, prepared in sterile water, without further sterilization. Suspensions were freshly prepared before adding them to the culture medium. All yeast derivatives and cells were newly added when the medium was refreshed every three days, throughout the full duration of biofilm cultivation. Three concentrations for each ingredient were used. These were 0.25% (low), 0.5% (mid), 0.75% (high) in healthy biofilms and 0.5% (low), 0.75% (mid), and 1.00% (high) in periodontitis biofilms. For live yeast (LY-B), 10^7^ (low), 10^8^ (mid), and 10^9^ (high) CFU/ml were used. The biofilm cultured in medium without any product was used as a negative control for all experiments (also in triplicates).

### Biofilm quantification

**Figure 1:**
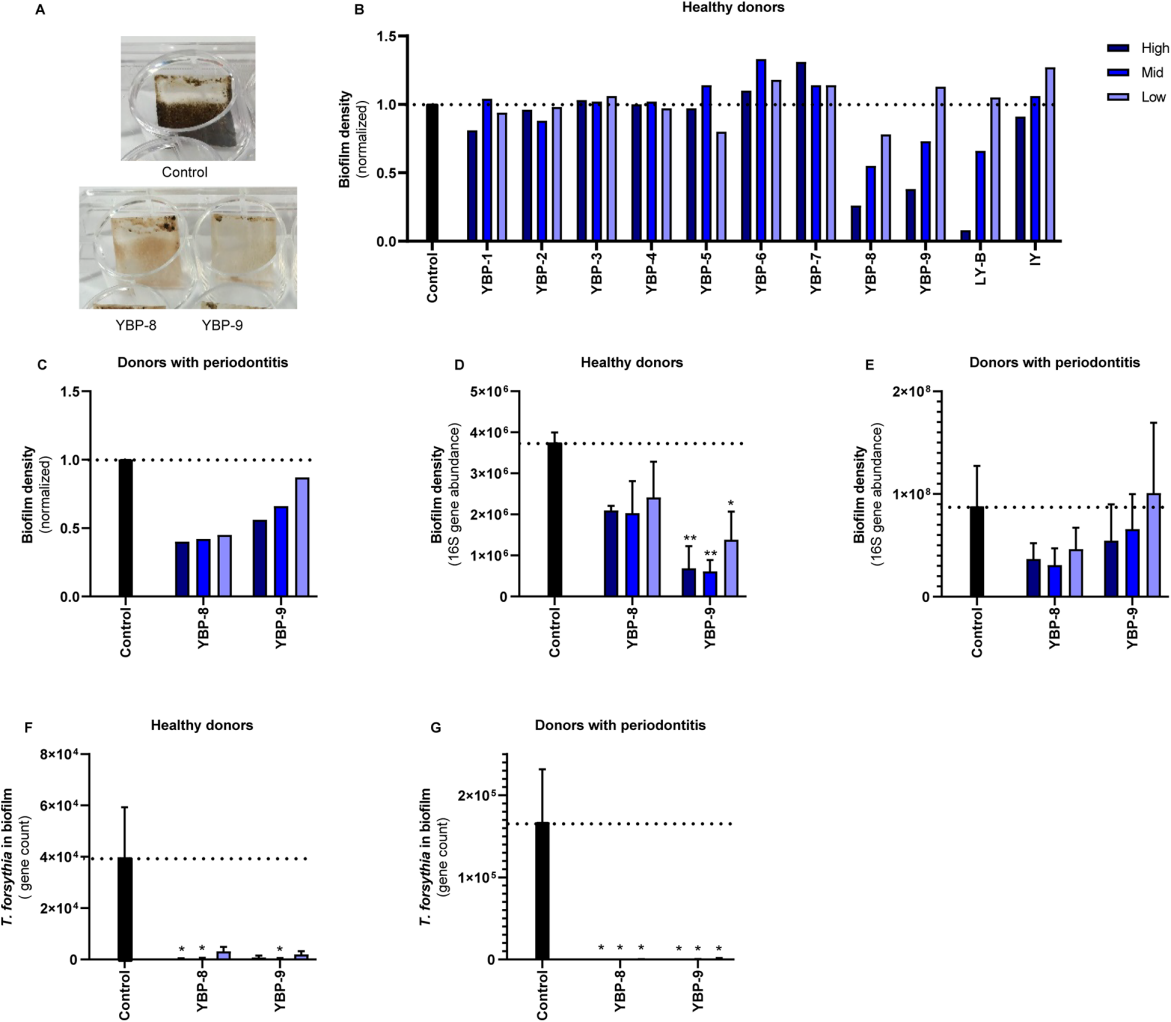
The effect of yeast products in the oral biofilm formation *in vitro*. **(A)** Illustrative images of the *in vitro* biofilm formation assay using oral microbiota from healthy donors. Both compounds were used at the highest concentration (0.75%). **(B)** Pooled oral microbiota from ten healthy donors was treated with different yeast products for nine days. Three concentrations for each ingredient were tested: 0.25% (low, light blue), 0.5% (mid, blue), 0.75% (high, dark blue) in healthy biofilms and 0.5% (low, light blue), 0.75% (mid, blue), and 1% (high, dark blue) in periodontitis biofilms. For live yeast (LY-B), 10^7^ (low), 10^8^ (mid), and 10^9^ (high) CFU/ml were used. This screening experiment was performed with single replicates; therefore, no statistics were performed. **(C)** Quantification of the biofilm formation of the experiments tested with YBP-8-9 using crystal violet staining of pooled oral microbiota from donors with periodontitis. **(D,E)** Quantification of the biofilm density with qPCR of the 16S gene of YBP-8-9. (F–G) Quantification of *Tannerella forsythia* in the biofilms of YBP-8-9. Statistics: Two-way ANOVA with Dunnett's multiple comparison, *p ≤ 0.05, **p ≤ 0.01. The mean with SEM of 3 independent experiments **(D–G)** is shown. Yeast-Based Products (YBP) Inactivated Yeast (IY), Live Yeast (LY-B).

Upon harvesting, photos were taken of the biofilms to monitor the biofilm appearance (Figure 1A and Supplementary Material S1). Biofilms were quantified by crystal violet staining. After harvesting, biofilms were fixed in 96% ethanol and stained with crystal violet (Pro-lab Diagnostics, Canada). Bound crystal violet was solubilized in 33% acetic acid. Optical density (OD) was measured at 580 nm on the BioTek Synergy Neo2 plate reader (Agilent, US). The OD values were normalized to the density of the control medium for each experiment. The biofilms stanned with crystal violet staining were not used for any further analyses.

### Quantitative PCR

The bacterial load of the biofilm was determined through broad-range quantitative polymerase chain reaction (qPCR) using primers listed in Table 1. The qPCR was performed in RT PCR master mix (Diagenode, Belgium) on an Applied Biosystems 7500 RT PCR system (Thermo Fisher, US) during 45 cycles of with a denaturation step at 95.0°C for 15 s and an annealing/elongation step at 60.0°C for 1 min. A standard curve was established by analysis of genomic DNA of targeted species and total bacteria using the 16s rDNA.

**Table 1:** Oligonucleotides used in qPCR analysis.

Target	Sequence 5’- 3’	Label	
16s rDNA	CGA AAG CGT GGG GAG CAA A		
	GTT CGT ACT CCC CAG GCG G		
	ATT AGA TAC CCT GGT AGT CCA	FAM	MGB
*Tannerella forsythia*	GACTGTCAGTTGCTAACAGGTAAAGCT		
	CCAACCTTCCTCACAGCTTACG		
	ACTCTGGCGGGACTG	FAM	MGB
*Porphyromonas gingivalis*	GCGCTCAACGTTCAGCC		
	CACGAATTCCGCCTGC		
	CACTGAACTCAAGCCCGGCAGTTTCAA	FAM	MGB
*Treponema denticola*	GCCTGGTGTGAAATCTACGAGC		
	CTCCGTGATTCAAGTCAAGCAGTA		
	CAACTCGTAAACTGCATTGG	NED	MGB

### Microbial composition analysis

Biofilm composition was determined by amplicon sequencing of the V4 hypervariable region of the 16S ribosomal gene as described previously (25). We used no data preprocessing for the alpha diversity analyses with Shannon index. For descriptive statistical analysis and visualization, we used principal component analysis (PCA) and PERMANOVA including centered log-ratio (CLR) transformation and removal of low-abundance species (threshold set at 0.01%). Differentially abundant species between the test conditions were determined by DESeq2 (26) using the biofilms grown in cultivation medium (blank) as a reference. Effect sizes were determined by logfoldchange and levels of abundance by baseMean visualized by heatmaps. Genus levels were used to assign the bacterial groups of the oral microbiota, and a higher taxonomic level (genus nomenclature followed by “sp.” or higher) was used if it was not possible to assign species classification to a 16S gene fragment. Significance was determined by an adjusted *p* value <0.05. Plots were constructed with ggplot2 (27) in R version 4.0.2. Phyloseq (28) and DESeq2 (26) together with the vegan (adonis2) package were used for the analyses.

### Analysis of short chain fatty acids

For the analysis of SCFAs, the cultivation medium (supernatant) was harvested at day nine and filter sterilized. SCFAs concentrations were determined by liquid chromatography–mass spectrometry (LC-MS). For this, a 50-fold dilution of samples and SCFA calibration standards was made. Deuterated SCFA (acetic, propionic, butyric, and valeric acid) were added as internal standards. Sample/internal standard mixtures were derivatized with 3-nitrophenylhydrazine. Next, samples were analyzed by LC-MS using reversed-phase chromatography with a Waters BEH C18 column (100 × 2.1 mm i.d., Waters, US) on a Waters Acquity H-Class UPLC system (Waters, US) and a Thermo Q-Exactive high-resolution mass spectrometer (ThermoFisher, US). A linear regression model was constructed from the standard calibration data. SCFAs concentrations in the samples were calculated by applying the regression model to the sample data.

## Results

### Cultivation and biofilm appearance

In this study, we investigated the potential benefic effects of yeast derivatives in the oral microbiota using an *in vitro* model for biofilm formation. Oral biofilms were collected from both healthy individuals and those diagnosed with periodontitis. Donor material was pooled per phenotype and cultured with or without compounds for nine days (Supplementary Material Figure S1). Three independent experiments were conducted per phenotype, involving 5–10 volunteers for each replicate. After the nine-day biofilm cultivation, photos were taken of the medium and biofilms (Figure 1A). Differences were observed in the biofilm density and appearance, with the yeast cell wall derivatives exhibiting lower biofilm densities compared to the media control.

### Biofilm density

Initially, the effect of all products was screened using the dental plaque biofilm collected from the healthy volunteers. To evaluate the effect of the yeast-based products in the bacterial biofilm formation and density, crystal violet staining was employed in all biofilms (Figure 1B). Growth in YBP-6 and −7 resulted in slightly higher optical density values than the media control. Exposure to YBP-8, YBP-9, and live yeast (LY-B) showed a concentration-dependent reduction in crystal violet optical density readings. This experiment was repeated using material from donors with periodontitis (Figure 1C). A 50%–60% reduction in biomass was observed upon exposure to YBP-8. YBP-9 exhibited a reduction in biofilm biomass (0.56, 0.66, and 0.87 normalized biofilm density). Based on these results the yeast derivatives YBP-8 and YBP-9 were selected for validation and further analyses using biofilm of periodontal and healthy volunteers.

To validate the results with YBP-8 and YBP-9, three independent experiments were conducted using different pooled donor materials. The total bacterial DNA of the biofilm was determined based on qPCR and the abundance of the 16s rRNA gene revealed lower density in the presence of YBP-8 and −9 in the biofilm from health donors (Figure 1D). The most pronounced effects were observed for YBP-9, which were statistically significant. YBP-9 is composed of 50% beta-glucans while YBP-8 with 25% beta-glucans (Supplementary Table 1). A similar trend was observed with periodontitis material (Figure 1E). However, the variation between the experiments was much larger, and thus, no statistical significance was observed. Overall, yeast cell wall derivatives reduced the biofilm density in healthy and periodontitis biofilms.

### Biofilm microbial composition

Using qPCR, we analyzed the bacterial levels of the periodontal pathogens *Tannerella forsythia*, *Porphyromonas gingivalis* and *Treponema denticola*. As the density values of *P. gingivalis* and *T. denticola* were extremely low in the inoculum samples of the healthy volunteers, no significant growth of these species was detected in the *in vitro* model. *P. gingivalis* was detected in the periodontitis samples in two of the three pools. *T. forsythia* was quantified in qPCR in the biofilm models, and its abundance was influenced by the products tested. Significantly lower *T. forsythia* levels were detected in the presence of YBP-8 and YBP-9 in the biofilm from both health and diseased volunteers (Figures 1F,G).

**Figure 2:**
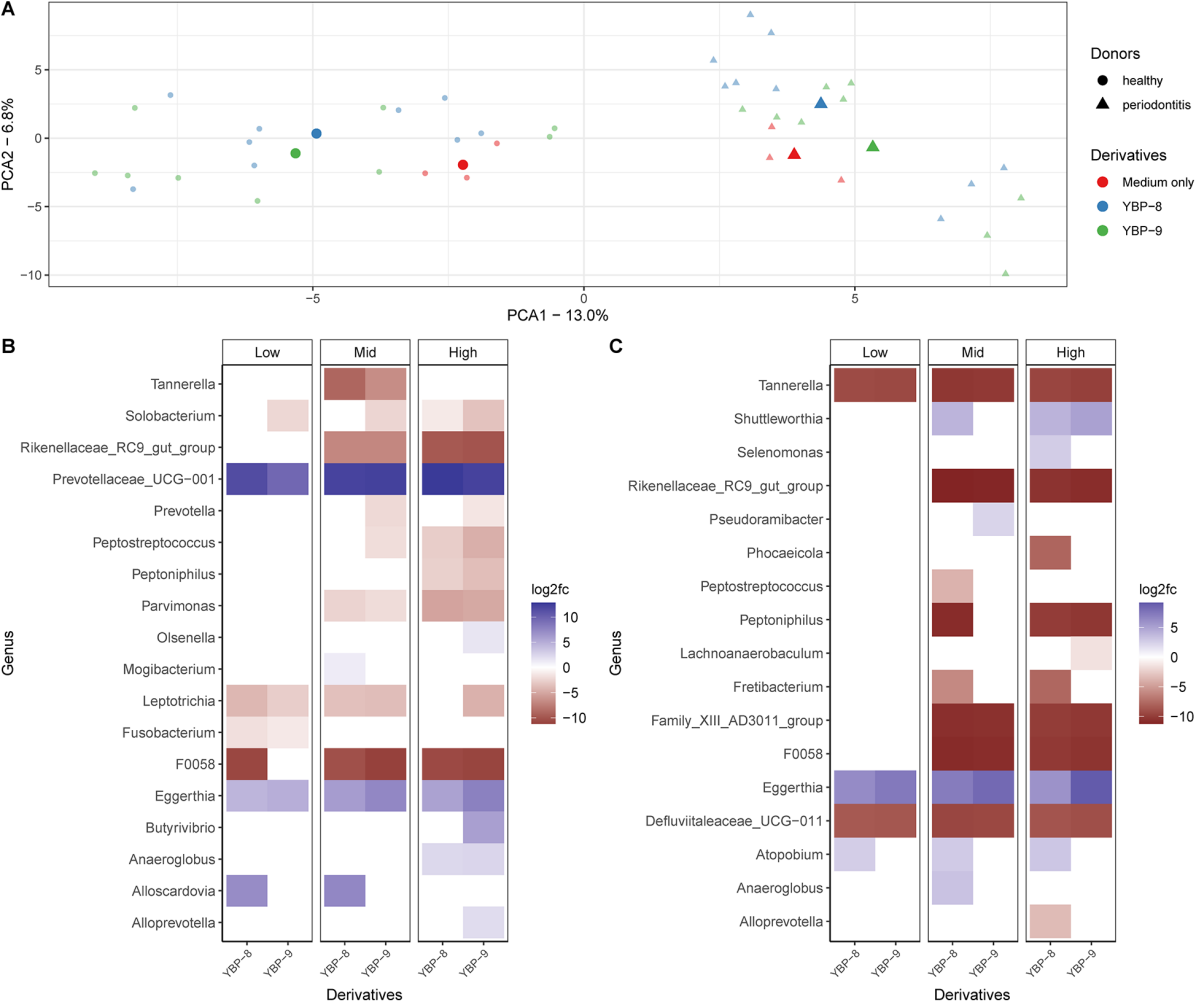
Effect of yeast cell wall derivatives in the oral biofilm *in vitro*. Three concentrations for each ingredient were tested: 0.25% (low), 0.5% (mid), 0.75% (high) in healthy biofilms and 0.5% (low), 0.75% (mid), and 1% (high) in periodontitis biofilms. **(A)** Multidimensional analysis of beta-diversity of healthy and periodontitis oral microbiotas after yeast cell wall extract treatment. In the heathy and periodontitis samples the two axes (PC1 and PC2) explain 13% and 6.8% of the total variation. The symbols represent single samples and when close together correspond to observations that have similar scores on the PCA components. Red represents all samples from the control without any product (medium only); blue are the biofilms treated with YBP-8; green are the biofilms tested with YBP-9. **(B)** Analysis of taxonomic abundances of healthy and **(C)** periodontitis biofilms with DESeq2 that were significantly different after treatment with YBP-8 and −9 (*p* < 0.05) on genus level. Taxonomic features which decreased in abundance after treatment are represented by a negative log2fc change, increased features have a positive log2fc change. Yeast-Based Products (YBP).

The 16S rDNA was sequenced to investigate the effects on the bacterial abundance. Similar to the targeted qPCR results, a significant reduction in the genus *Tannerella* was observed (Figure 2), particularly in the periodontitis donor material (Figure 2C). These results indicate that yeast cell wall derivatives also reduce the density of specific oral pathogens in biofilms.

Based on the sequencing results, significant changes in the composition and diversity of the microbiota were observed. The principal component analysis (PCoA) reveals significant differences between the pool of the donor materials—healthy and periodontitis (Figure 2A, *p* = 0.001). Furthermore, yeast cell wall derivatives also altered the biofilm composition (*p* = 0.001) and increased the diversity of the microbiome (Supplementary Material Figure S2A). These results suggest that yeast cell wall extracts do affect the biofilm composition.

The effects of yeast cell wall derivatives (YBP-8 and −9) on the abundance of specific genera differed between the two donor materials (Figures 2B,C). For example, *Leptotrichia* significantly decreased in the healthy donor material for both components. This genus was not significantly affected in the periodontitis material. On the other hand, *Eggerthia* significantly increased in both donor materials and for both compounds. These results suggest that yeast cell wall derivatives have differential effects on particular genera, potentially influenced also by the initial abundance of the bacteria. However, most significant changes are related to genera with low abundance in the oral biofilm (Figures 2B,C). The most abundant genera, such as *Veillonella*, *Streptococcus*, *Prevotella*, and *Bacteroides* showed trends, but were statistically not significant (Supplementary Material Figure S2B). A reduction of *Bacteroides* in healthy and periodontitis biofilm was observed with the increase of the concentrations of the yeast cell wall derivatives, similar trend for the *Streptococcus* genus was observed in the biofilm of healthy individuals. The abundance of *Veillonella* and *Prevotella* remained stable or had a slight increase.

Treatment with yeast extracts (YBP-1 to −7) had minor effects on the microbiome composition (Supplementary Material Figure S3). YBP-1, YBP-2, and YBP-6 might reduce the bacterial diversity; however, it is not statistically significant (Supplementary Material Figure S3A). There was no effect on either biofilm formation, or composition expected from these compounds. On the contrary, YBP-3, YBP-5, and YBP-7 might increase the diversity of the microbiome, however, not statistically significant (Supplementary Material Figure S3A). Overall, there was no significative effect on the composition of the microbiota. Interestingly, even though not statistically significant, both YBP-3 and YBP-7 increased the saccharolytic commensal *Streptococcus,* in addition, was also observed an increase on the abundance of *Veillonella* species compared to the control (Supplementary Material Figure S3B).

### Short chain fatty acids (SCFAs)

**Figure 3:**
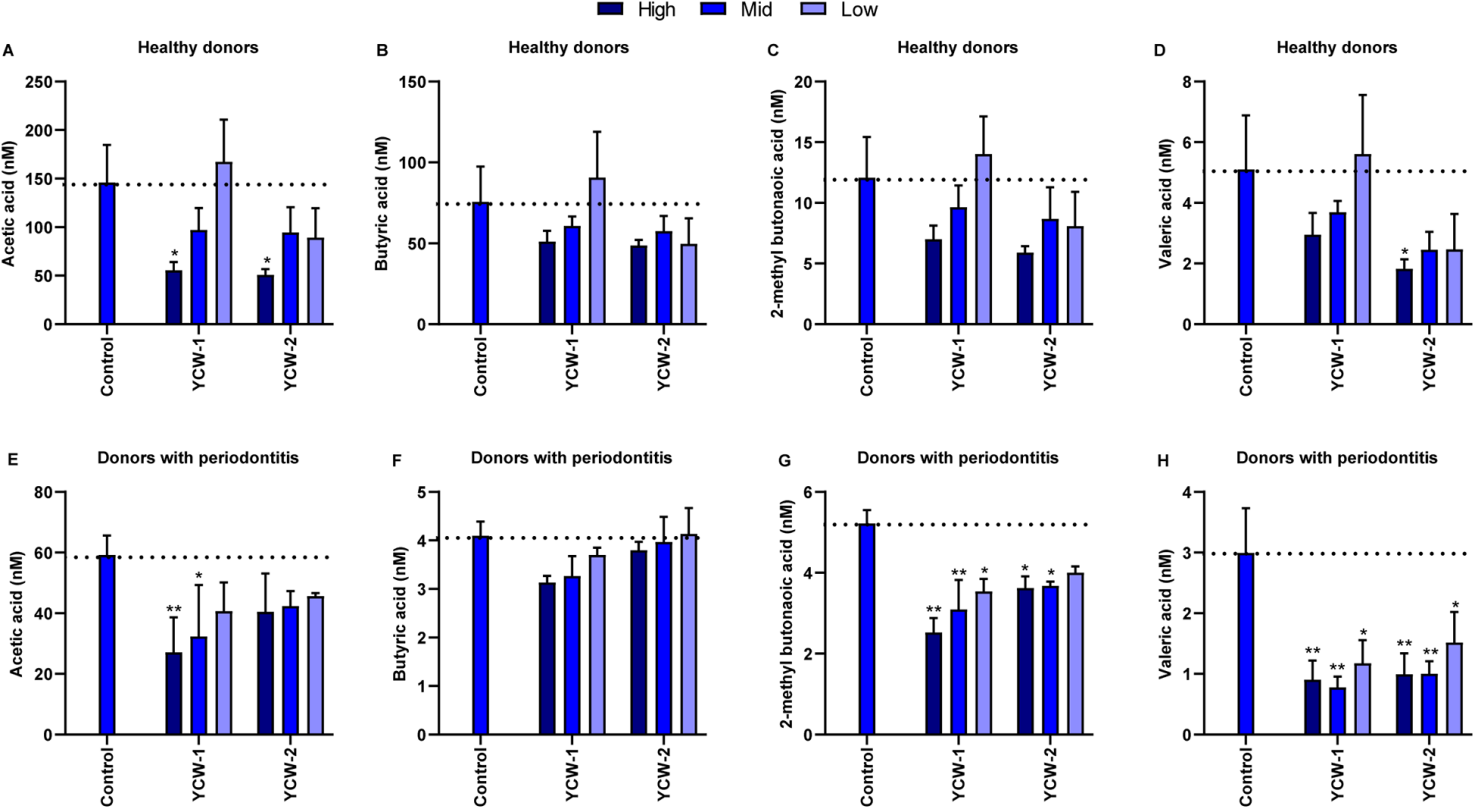
Yeast cell wall derivatives decrease short chain fatty acids (SCFAs) production *in vitro*. Three concentrations for each ingredient were used: 0.25% (low), 0.5% (mid), 0.75% (high) in healthy biofilms **(A–D)** and 0.5% (low), 0.75% (mid), and 1% (high) in periodontitis biofilms **(E–H)**. SCFAs were measured in supernatants of oral microbiome cultures. Statistics: Two-way ANOVA with Dunnett's multiple comparison, *p ≤ 0.05, **p ≤ 0.01. The mean with SEM is shown. Yeast-based products (YBP).

SCFAs are produced through bacterial fermentation and were quantified from the used medium (supernatants) of each experiment on day nine in both donor (healthy and periodontitis) derived biofilms (Figure 3 and Supplementary Material S4). When compared to the control condition, exposure to YBP-8 and YBP-9 resulted in a lowering of the production of acetic acid levels in the biofilms (Figures 3A,E). Even though it was not statistically significant, a reduction in the concentration of butyric acid was observed due to the exposure to yeast cell wall extracts (Figures 3B,F). In the periodontitis derived biofilm, the yeast cell wall derivatives significantly decreased 2-methyl butanoic acid and valeric acid (Figures 3G,H), which was only partly significant in biofilms from healthy individuals (Figures 3C,D). Overall, the tested compounds have an effect on the oral microbiota composition and function.

## Discussion

The disruption of a stable oral microbiome ecology often results in the overgrowth of low abundance pathobionts, leading to an increased incidence of oral diseases, such as periodontitis. This condition is highly influenced by poor oral hygiene and biofilm formation (1). To address this problem, a variety of agents have been formulated into oral care products to improve their efficacy in controlling dental plaque (25). Inactivated yeast cells, yeast extracts, and yeast cell walls are classified as both prebiotics and postbiotics due to their ability to supply substrates for host microorganisms and might support health benefits. On the other hand, live yeast cells function as probiotics (29). In the present study, we evaluated the effects of eleven yeast-based products, including pre-, post-, and probiotics, on an *in vitro* oral biofilm model. Biofilms were cultured from dental plaque samples collected from both healthy individuals and those diagnosed with periodontitis. Among the compounds tested, the yeast cell wall derivatives had a more pronounced impact on the oral biofilm compared to the yeast extracts. During the cultivation of the oral biofilms, it was evident that the exposure to yeast cell walls had a significant influence on the biofilm's appearance and composition.

The metabolism of microorganisms within the biofilm creates a complex microenvironment, characterized by inter- and intra-species interactions, as well as the exchange of nutrients, fermentation products, and factors influencing pH and redox potential (26). Certain molecules, such as heme, can activate signaling pathways that lead to the synthesis of various extracellular components. In general, pathogenic bacteria require the iron-containing cofactor heme to express virulence and resistance phenotypes (27). The darker color observed in the control biofilm may suggest higher levels of heme, indicative of increased hemolytic activity, compared to the biofilms treated with the yeast-based derivatives (28). The lighter color of the biofilms after treatment with the yeast derivatives YBP-8 and YBP-9 may indicate lower levels of heme, potentially promoting the growth of commensal bacteria.

A relevant reduction in biofilm density was observed after exposure to yeast cell wall derivatives, as assessed using crystal violet and by determining the total bacterial DNA load of the biofilm. The total bacterial and specific oral pathogens’ density, such as *T. forsythia*, were reduced. Reducing biofilm formation and the abundance of oral pathogens are well-accepted strategies for preventing oral diseases (29).

It was also observed that the yeast cell wall derivatives significantly changed the composition of the microbiome *in vitro*. Both YBP-8 and YBP-9 reduced the abundance of *Leptotrichia* and increased *Eggerthia* in healthy donor biofilm. *Leptotrichia* is often found in the filament-rich annulus of dental biofilms, suggesting its potential importance in structuring dental plaque communities during maturation (30). *In vitro* studies have shown that *Eggerthia* is enriched in protein and serum-rich media (31), suggesting it may play a role in protein metabolism within the oral biofilm. This indicates that in a healthy, balanced oral ecosystem, *Eggerthia* could have a non-pathogenic function as part of the commensal microbiome. However, while *Eggerthia* may have a neutral or even beneficial role in oral biofilms, it may become more problematic or opportunistic in the context of periodontal disease. There is evidence which suggests that an increase in the abundance of *Eggerthia* is positively correlated with the severity of periodontal disease, as measured by mean probing depth (MPD) (30). Its increased prevalence during periodontal disease may be a consequence of the altered environmental conditions and microbial interactions, rather than a direct causative factor. This association implies that the relationship between *Eggerthia* and oral health is complex and not yet fully elucidated. The exposure of yeast cell wall derivatives (YBP-8 and-9), even though not statistically significant, also showed an increase in *Veillonella* species, which are known for their ability to metabolize lactate, helping to reduce the acidity in the oral cavity (32). These results suggest that these two yeast cell wall derivatives may not directly affect the biofilm formation but rather its composition.

Yeast cell wall components, particularly polysaccharides like β-glucans and mannans, have shown significant potential in combating biofilm formation. β-glucans and mannans can prevent the initial adhesion of bacteria to surfaces by blocking adhesion sites or modifying the surface properties, which is a critical first step in biofilm formation (33). By participating in bacterial aggregation, the insoluble yeast cell wall material may directly compete with the binding of bacteria onto the biofilm. The antibiofilm properties of β-glucans and mannans derived from yeast cell walls offer a promising strategy to combat biofilm-associated infections (34, 35). However, we noticed slight differences between the different compounds tested here. Growth conditions and different strains might have an influence on the effect of the oral biofilm as it has been shown to affect composition and chemical structure of the preparations (31–33).

Previous studies have demonstrated the ability of yeast-derived compounds, such as mannoproteins, to influence biofilm development. For instance, mannoprotein from the yeast *Saccharomyces cerevisiae* has been shown to stimulate or inhibit the growth of both Gram-positive and Gram-negative bacteria, thereby affecting biofilm formation (34). Additionally, Walencka et al. reported that a crude extract from the cell wall of *S. cerevisiae*, containing mannoprotein with surfactant activity, was able to reduce biofilm growth, alter biofilm morphology, and accelerate the detachment of mature biofilms of *Staphylococcus aureus* (35).

In the present study, we observed that the yeast extracts (YBP-3 and YBP-7) did not significantly alter the overall biofilm density, but they exhibited distinct modulatory effects on the biofilm composition. Notably, exposure to both YBP-3 and YBP-7 resulted in a higher relative abundance of *Streptococcus* species. This may suggest that the tested yeast extracts have prebiotic and postbiotic-like effects, supporting the growth of commensal streptococci, which are known to play a crucial role in the formation and structure of dental biofilms. Commensal streptococci are among the first colonizers of the tooth surface and are essential for the formation of dental biofilms (36). Some *Streptococcus* species can produce secondary metabolites with antimicrobial activity, inhibiting the colonization and growth of pathogenic bacteria, as well as directly influencing the microbial diversity of the oral cavity (36). However, this genus is also crucial for oral carbohydrate metabolism, which can lead to an increase in acid production and potential tooth decay (37), highlighting the ambivalent role of *Streptococcus* in oral health. Nevertheless, a larger number of *Streptococcus* species have been explored as probiotics for oral health due to their ability to positively alter the structure of the oral microbiome, supporting several ecological interactions between commensals and pathogens (36).

The short-chain fatty acids (SCFAs) produced by the oral microbiota, play a significant and multifaceted role in oral health due to their influence on the microbiome composition, immune response, and local tissue. Unlike their beneficial role in intestinal health, SCFAs in the oral cavity, such as acetate and butyrate, may have negative influence in the health of the oral environment. SCFAs can be used as an energy source by certain oral pathogens, thus promoting their proliferation (38). High concentrations of SCFAs in the oral cavity are often due to an increase in the fermentation of carbohydrates and proteins by cariogenic bacteria, which contributes to the demineralization of tooth surfaces and the development of oral diseases (38). However, the concentration of SCFAs is only one factor among several others which may promote the development of oral diseases, such as dental caries. The frequency and duration of exposure to the acids, as well as the buffering capacity of saliva and the overall balance of the oral microbiome, also play crucial roles in the caries process.

Interestingly, the yeast cell wall derivatives in this study reduced the production of several types of SCFAs, particularly acetate. Furthermore, the yeast cell wall derivatives reduced the levels of 2-methyl butanoic acid and iso-butyric acid, suggesting a potential impairment of leucine fermentation capacity in the biofilm (39). Amino acid fermentation has been associated with periodontitis (34), although the *in vivo* effects have not been sufficiently established, and this area remains an active area for further investigation. The reduction in SCFA production observed in this study might not necessarily be related to a direct inhibition by the yeast cell wall derivatives, but rather an antimicrobial effect that lowers the overall biofilm density. These findings indicate a positive effect of the yeast cell wall derivatives in potentially lowering the amount SCFAs which can participate in the development of local oral diseases and potential dysbiosis.

It is clear from our findings that the yeast cell wall derivatives had a significant impact on biofilm activity, but the exact mechanism of action remains unclear. Even though we did not observe a direct antimicrobial effect of the yeast cell wall derivatives, the exposure led to changes in the fermentation of these derivatives, causing compositional and functional changes, such as lowering biofilm biomass and altering the SCFA and microbiota profiles. The ability of yeast cell wall derivatives and their components, such as β-glucans and mannans, to inhibit biofilm formation, disrupt established biofilms, modulate immune responses, and enhance efficacy of antimicrobials combining with their antagonistic properties, positions them as valuable and broadly applicable strategies to promote oral health (22, 34, 35).

In conclusion, this study has demonstrated the potential of yeast cell wall derivatives, such as YBP-8 and YBP-9, to positively impact the oral biofilm and microbiome. The exposure to these yeast-based derivatives resulted in significant changes, including reduced biofilm density, altered composition of the microbial community, and modulation of metabolic activities like the SCFA production. The observed reduction in biofilm formation and the abundance of certain oral pathogens, such as *Tannerella forsythia*, suggests that yeast cell wall derivatives could be a promising strategy to combat biofilm-associated oral diseases. This compounds’ ability to potentially inhibit bacterial adhesion, disrupt established biofilms, and influence the balance of the oral microbiome is noteworthy. While the exact mechanisms behind the effects of yeast cell wall derivatives on the oral biofilm remain to be fully elucidated, the findings of this study provide valuable insights. The reduction in SCFA production, particularly of compounds associated with amino acid fermentation, and the modulation of key bacterial genera like *Leptotrichia* and *Eggerthia*, indicate that these yeast-based products can have a significant impact on the metabolic activities and ecological dynamics within the oral biofilm. Overall, the results of this study highlight the potential of yeast cell wall derivatives as a novel approach to promoting oral health by targeting the complex interplay between biofilm formation, microbial composition, and metabolic processes in the oral cavity. Further research is warranted to fully understand the underlying mechanisms and to explore the clinical applications of these yeast-based products in the management of oral diseases.

## Data Availability

The original data presented in the study are publicly available. This data can be found in the ENA database from EBI: PRJEB85284.
