# Supplementary material for: Yeast cell wall derivatives as a potential strategy for modulating oral microbiota and dental plaque biofilm
Source: Front Oral Health. 2025 Feb 13;6:1543667. doi: 10.3389/froh.2025.1543667 (PMC11865069; doi:10.3389/froh.2025.1543667)
Supplement: Supplementary file 1 [file Datasheet1.docx]

Supplementary Material

# Supplementary Figures


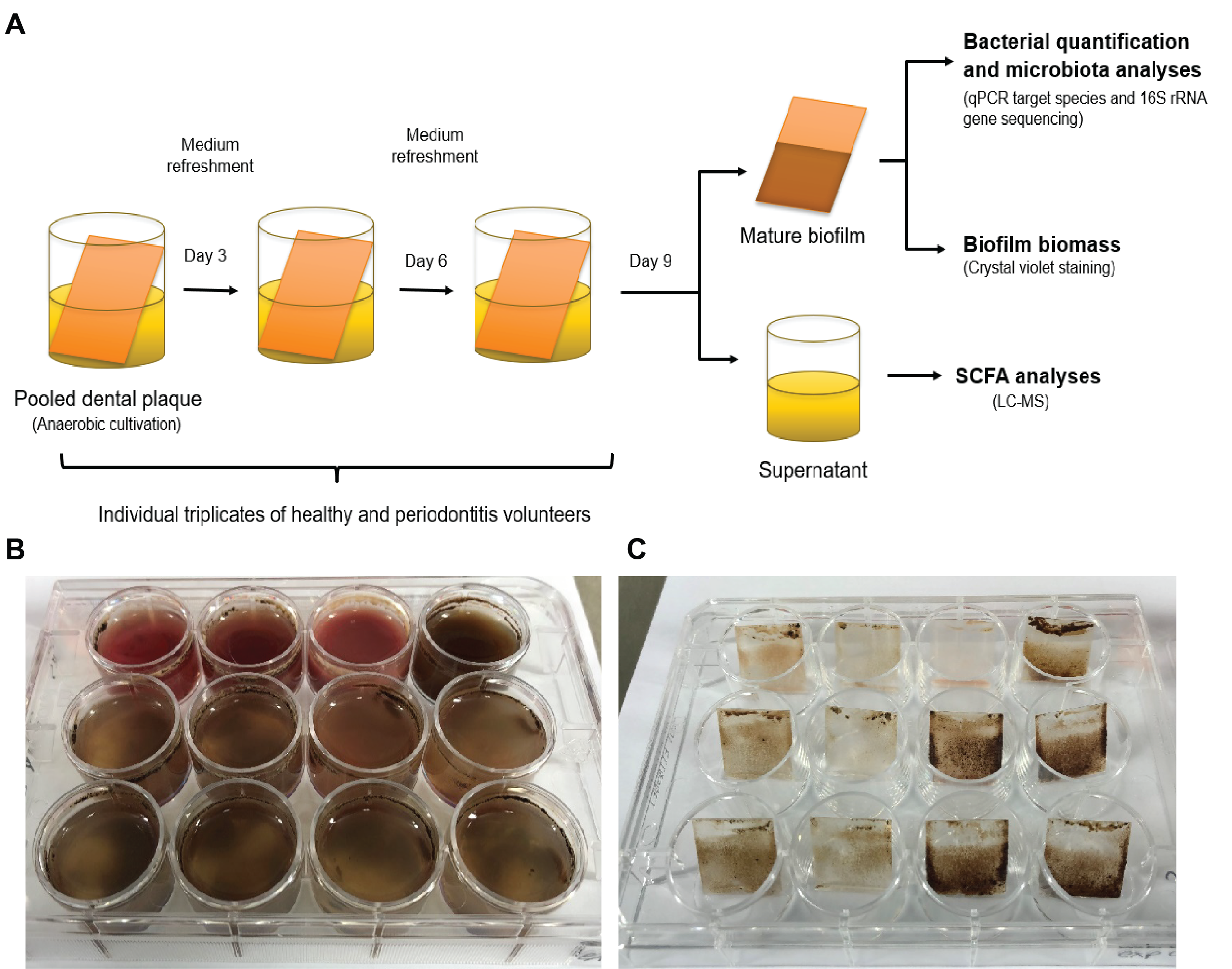


**Supplementary Figure 1** Overview of oral microbiome culture *in vitro*. (A) Schematic overview of the procedure and measurements. (B) Example of cultured oral microbiome and (C) slides used for biofilm formation.


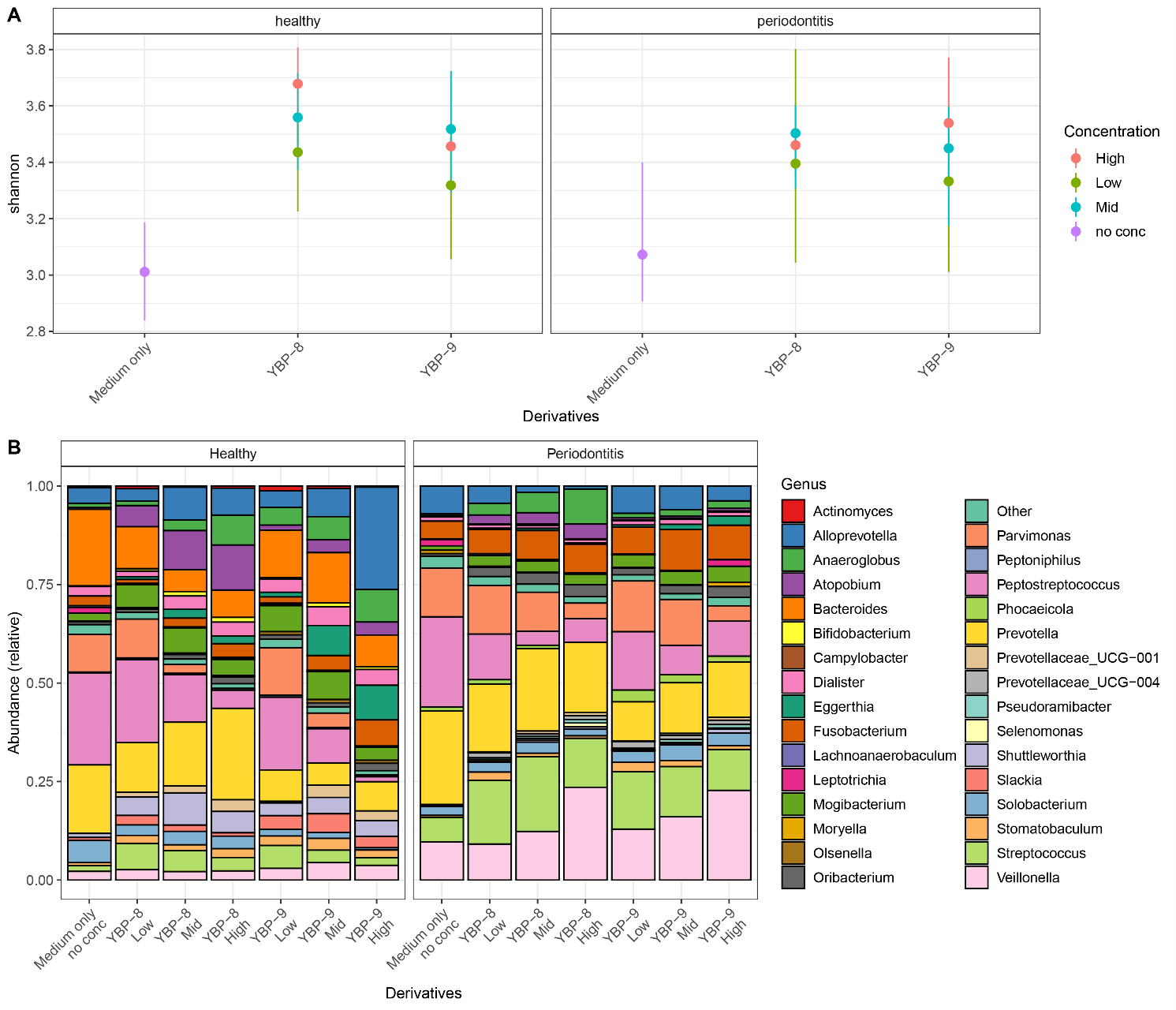


**Supplementary Figure 2.** Yeast cell wall derivatives change the composition of the oral microbiome *in vitro*. Healthy donor microbiomes and periodontitis microbiomes are shown. (A) Alpha diversity of the oral microbiome after treatment with different yeast derivatives. (B) Relative abundance of the most abundant genera. Abbreviations: Yeast-Based Products (YBP).


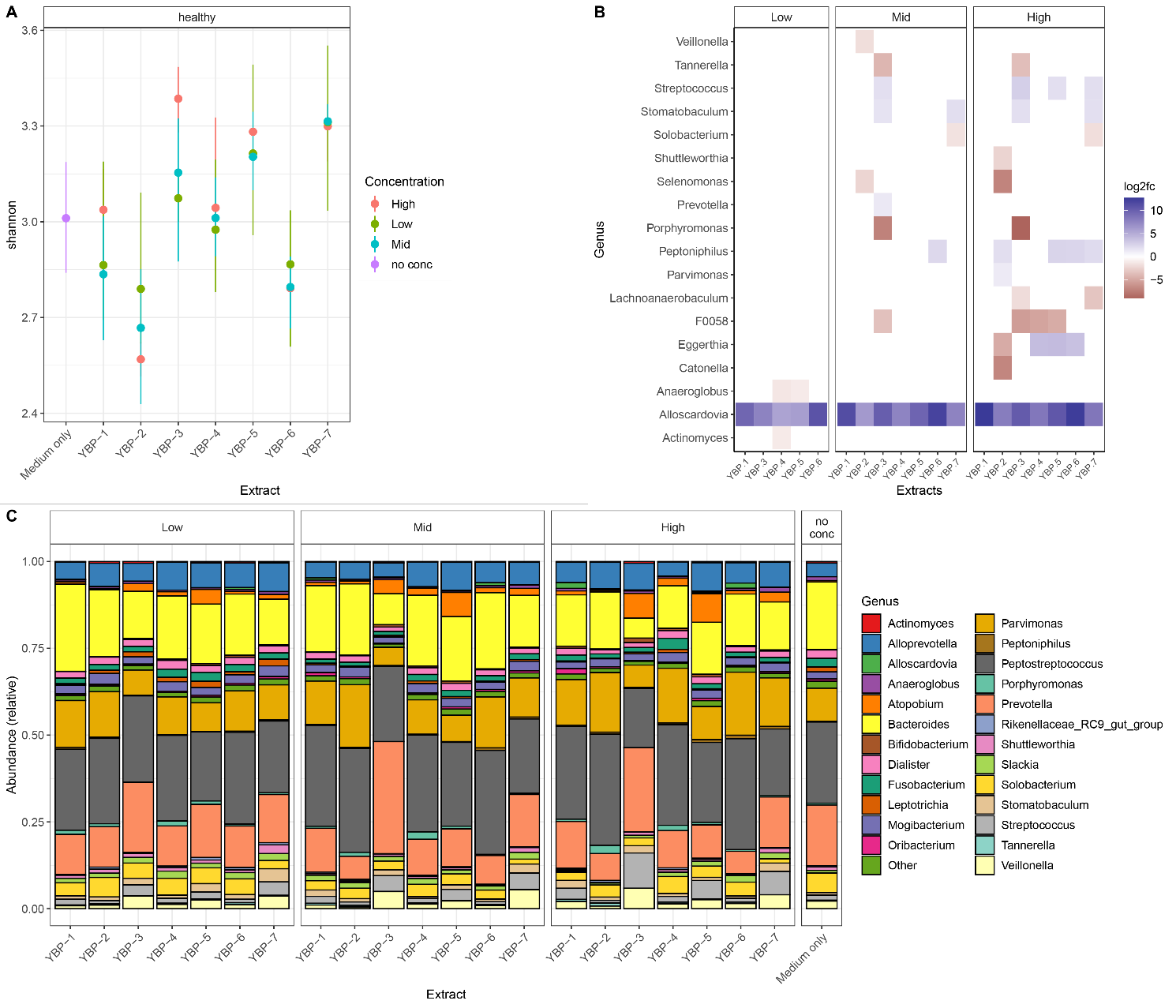


**Supplementary Figure 3.** Yeast extracts change the composition of the oral microbiome from healthy subjects *in vitro*. (A) Alpha diversity of the oral microbiome after treatment with different yeast extracts. (B) Relative abundance of the most abundant genera. (C) Significantly changed genera after treatment. Abbreviations: Yeast-Based Products (YBP).


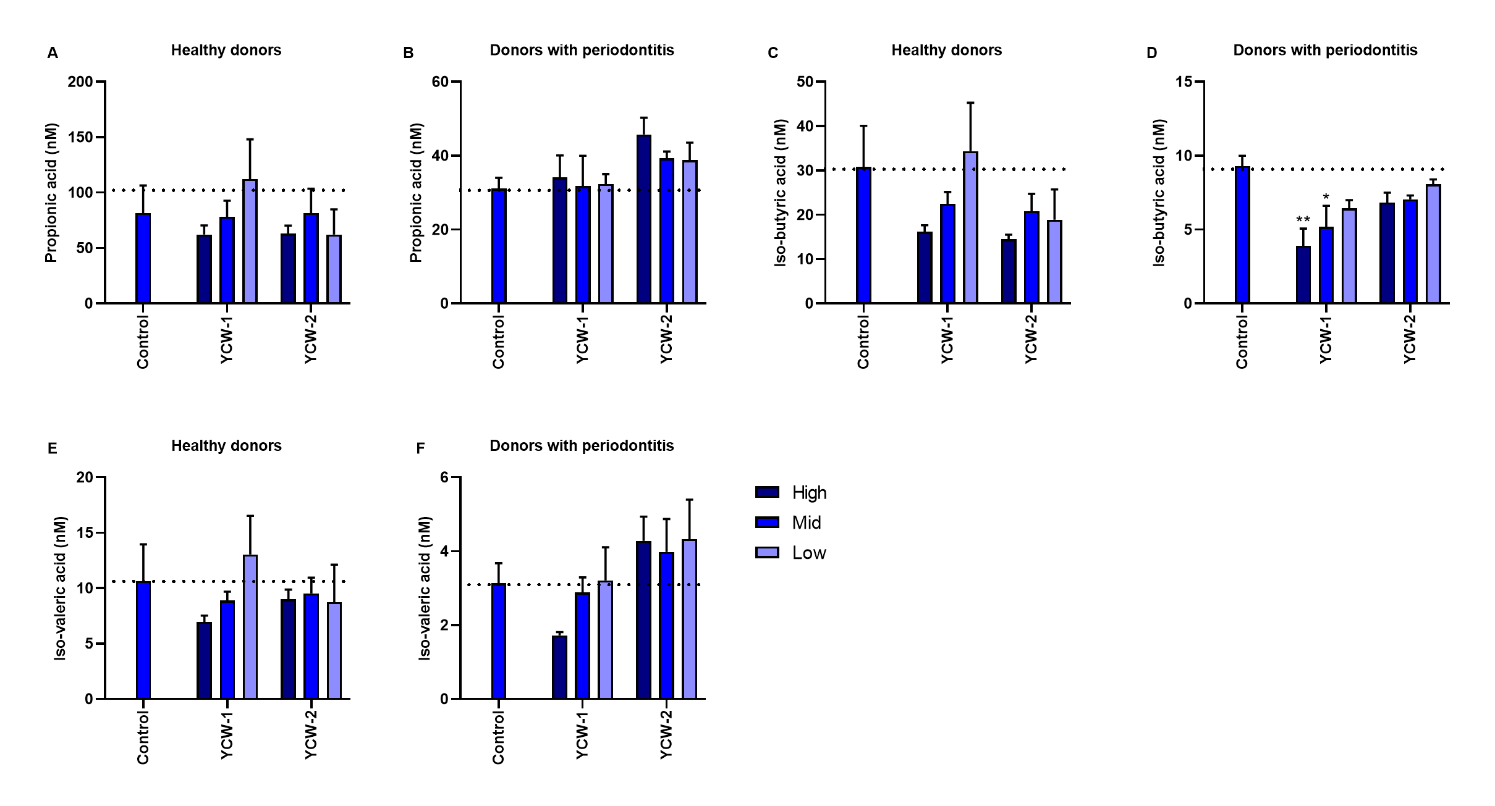


**Supplementary Figure 4.** Yeast cell wall derivatives decrease short chain fatty acids production *in vitro*. (A, B) Concentrations of propionic acid. (C, D) Iso-butyric acid, and (E, F) Iso-valeric acid of either healthy microbiomes or from donors with periodontitis. Statistics: Two-way ANOVA with Dunnett’s multiple comparison, *p≤0.05, **p≤0.01. The mean with SEM of 3 independent experiment is shown. Abbreviations: Yeast-Based Products (YBP).

# Supplementary Tables

**Supplementary Table 1.** Yeast-based products used in this study. The origin, composition and supplier are described for each product.

| **Product#** | **Origin and composition**  *(Expressed on product as is)* | **Supplier** |
| --- | --- | --- |
| YBP-1 | YBP-1 is a yeast extract obtained from a selected baker’s yeast strain.  Proteins 56-63 g/100 g (Nitrogen x 6.25)  pH 5.5-6.1  Dry matter min. 94 g/100g | Biospringer by Lesaffre |
| YBP-2 | YBP-2 is a yeast extract obtained from a selected baker’s yeast strain.  Proteins 63-68 g/100 g (Nitrogen x 6.25)  pH 5.3-5.9  Dry matter min. 94 g/100g | Biospringer by Lesaffre |
| YBP-3 | YBP-3 is a yeast extract obtained by autolysis of brewer's yeast.  Proteins min. 58 g/100 g  Total nitrogen min. 9.2 g/100g  pH 5.4-6.2  Dry matter min. 94 g/100g | Procelys by Lesaffre |
| YBP-4 | YBP-4 is a yeast peptone obtained by the autolysis of a selected strain of baker’s yeast.  Proteins min. 62.5 g/100 g  Total nitrogen min. 10 g/100g  pH 6.2-7.2  Dry matter min. 94 g/100g | Procelys by Lesaffre |
| YBP-5 | YBP-5 is a yeast extract obtained by the autolysis of a selected strain of baker’s yeast.  Proteins min. 62.5 g/100 g  Total nitrogen min. 10 g/100g  pH 6.8-7.2  Dry matter min. 94 g/100g | Procelys by Lesaffre |
| YBP-6 | YBP-6 is a yeast extract obtained by the hydrolysis of a selected strain of baker’s yeast.  Proteins min. 63 g/100 g  Total nitrogen min. 10.1 g/100g  pH 5.5-6.5  Dry matter min. 94 g/100g | Procelys by Lesaffre |
| YBP-7 | YBP-7 is a yeast extract obtained from a selected strain of baker’s yeast  Proteins 46.9 - 56.3 g/100g  Total nitrogen 7.5 - 9.0 g/100g  pH 6.3-6.7  Dry matter min. 94 g/100g | Biospringer By Lesaffre |
| YBP-8 | YBP-8 is a yeast cell wall gently extracted from *Saccharomyces cerevisiae.*  Β-glucans 23-29 g/100g  Mannans 20-25 g/100g  Dry matter min.94 g/100g | Gnosis by Lesaffre |
| YBP-9 | YBP-9 is a purified yeast fraction rich in beta-glucans (1,3 and 1,6), obtained from primary grown proprietary strain of *Saccharomyces cerevisiae*.  β-1,3/1,6-glucans min. 50 g/100g  Dry matter min. 94 g/100g | Phileo by Lesaffre |
| LY | LY is an active dried yeast *Saccharomyces cerevisiae var. boulardii* CNCM I-3799 (commonly named *Saccharomyces boulardii*)  Culturable yeast cells min. 2x10^10^ CFU/g  Dry matter 94,5-96,5 g/100g | Gnosis By Lesaffre |
| IY | IY is an inactivated dried yeast obtained by drum-drying of *Saccharomyces cerevisiae*, primary grown on a molasses-based media  Proteins 46,8-55 g/100g  Dry matter min 94 g/100g | Gnosis By Lesaffre |
